# Molecular typing of PVL-positive *Staphylococcus aureus* isolates from Lithuania and genome sequencing of a local outbreak strain

**DOI:** 10.1038/s41598-026-61973-x

**Published:** 2026-07-18

**Authors:** Stefan Monecke, Elke Müller, Agnė Kirkliauskienė, Lilian J. B. Schneider, Maksim Bratchikov, Sascha D. Braun, Celia Diezel, Katrin Frankenfeld, Jolanta Miciulevičienė, Martin Reinicke, Lukas Rüttinger, Greta Vizujė, Hans-Hermann Söffing, Ralf Ehricht

**Affiliations:** 1 Leibniz-Institute of Photonic Technology (Leibniz-IPHT), Jena, Germany; 2 Jena University Hospital, Friedrich Schiller University Jena, Jena, Germany; 3 InfectoGnostics Research Campus, Centre for Applied Research, Jena, Germany; 4 Center for Translational Medicine (CETRAMED), Jena University Hospital, Friedrich Schiller University Jena, Jena, Germany; 5 Department of Physiology, Biochemistry, Microbiology, and Laboratory Medicine, Institute of Biomedical Sciences, Faculty of Medicine, Vilnius University, Vilnius, Lithuania; 6 Research Center for Medical Technology and Biotechnology, INTER-ARRAY, part of fzmb GmbH, Bad Langensalza, Germany; 7 Laboratory Medicine Centre, Vilnius City Clinical Hospital, Vilnius, Lithuania; 8 Senova Gesellschaft für Biowissenschaft und Technik mbH, Weimar, Germany; 9 Microbiology Laboratory, Republican Vilnius University Hospital, Vilnius, Lithuania; 10 Institute of Physical Chemistry, Friedrich Schiller University Jena, Jena, Germany

**Keywords:** Diseases, Microbiology, Molecular biology

## Abstract

*Staphylococcus aureus* is a major human commensal and pathogen, with the Panton-Valentine leukocidin (PVL) genes being associated with increased virulence. Rapid detection and molecular typing of such isolates are essential for effective epidemiological surveillance and infection control. Studies from Lithuania indicate that PVL is relatively common among *S. aureus* isolates, but detailed molecular typing data are still lacking. Clinical *S. aureus* isolates were collected from two hospitals in Vilnius in 2018–2019 and 2024, and from healthy volunteers between 2012 and 2020. Isolates were screened for PVL genes using real-time PCR. In addition, positive isolates harvested directly from the agar plate were tested for PVL production using an experimental lateral flow assay (LFA). Positive isolates were characterised using DNA-microarrays that facilitated the detection of resistance markers and virulence genes including PVL as well as an assignment to clonal complexes, strains and SCC*mec* types. Epidemiologically relevant isolates were subjected to whole-genome sequencing using Oxford nanopore technology. Out of 1296 *S. aureus* isolates, 124 yielded PVL-positive PCR results. 100 isolates were available for genotyping. Two PCR-positive isolates were negative by array and LFA, but PVL detection by DNA-microarray and lateral flow test (LF) showed complete concordance. Among PVL-positive isolates, 61.2% were methicillin-resistant *S. aureus* (MRSA). The most common PVL-MRSA strain (*n* = 43) was a Clonal Complex (CC) 8 MRSA that resembled the North American “USA300” strain but that lacked the arginine catabolic mobile element (ACME). This suggested an outbreak in one of the participating hospitals. Other common strains were PVL-positive CC30-MSSA, CC121-MSSA and CC8-MRSA-[IV+ACME] “USA300”, while other lineages were represented by single isolates only. Whole-genome sequencing of two ACME-negative CC8-MRSA-IV isolates and of one local “USA300” isolate, as well as a comparison to international reference sequences, showed a very high degree of similarity in core genome and prophage content. The dominant PVL-MRSA strains were “USA300”, indicating a possible importation from North America, and a locally emerged variant of “USA300” that lost the ACME-associated genes of its SCC*mec* element. These findings suggest that real-time PVL detection is essential for outbreak prevention. Given its clinical relevance, routine PVL screening —via PCR or lateral flow assays—in routine diagnostics should be seriously considered and surveillance of PVL-MRSA is urgently recommended.

**Supplementary Information:**

The online version contains supplementary material available at 10.1038/s41598-026-61973-x.

## Introduction

*Staphylococcus aureus* (*S. aureus*) is a Gram-positive coccus that colonises the skin and mucous membranes of humans and animals. Beyond asymptomatic colonisation, it can cause a wide variety of skin and soft tissue infections (SSTI, including abscesses, furunculosis, cellulitis, mastitis and necrotising fasciitis), pneumonia, sepsis, endocarditis, osteomyelitis, Staphylococcal toxic shock and scalded skin syndromes, as well as food intoxications. It harbours a wide array of virulence-associated genes, many of which reside on mobile genetic elements (MGEs) that include phages/prophages, plasmids, pathogenicity islands and others. *S. aureus* has also acquired a wide variety of genes causing resistance to antimicrobial agents. The first strains that carried penicillinase emerged already in the 1940s, shortly after penicillin entered general clinical use. The clinically most relevant resistance genes, however, are *mecA* and *mecC* encoding modified penicillin-binding proteins that confer resistance to oxacillin/methicillin (hence, MRSA for methicillin-resistant *S. aureus*) and to virtually all beta-lactam antibiotics, except for ceftobiprole and ceftaroline that have recently been developed to target MRSA. The *mec* genes are localised on complex MGEs, called staphylococcal cassette chromosome elements (SCC*mec*). Based on their structural organisation and accessory gene content, SCC*mec* elements have been classified into distinct types^1^. The older classes of SCC*mec* elements (I to III) appear to be largely restricted to hospital settings which has been attributed to adverse effects on fitness and growth rates compared to susceptible wild-type strains^2,3^. The more recently evolved SCC*mec* elements of types IV and V can also be found in community settings and livestock. SCC*mec* IV and V strains also gained a foothold in healthcare settings other than hospitals (such as rehabilitation centres or nursing homes) and from there, they colonised hospitals largely displacing and marginalising the older SCC*mec* I to III strains. In some parts of the world, especially the Middle East, North Africa and New Zealand, composite elements comprising SCC*mec* types IV or V plus the fusidic acid resistance gene *fusC* with a set of surrounding genes^4^ have emerged as a significant concern, being detected in both hospital and community settings.

One virulence factor that often plays a role in the development of SSTIs and pneumonia is Panton-Valentine leukocidin (PVL^5–7^) . It consists of two components encoded by two adjacent genes located on a prophage (*lukS-PV*,* lukF-PV*, GenBank BA000033.2:MW1378 and MW1379^8–10^). Together, these proteins form polymeric pores in leukocyte membranes, inducing cell lysis, apoptosis and a strong inflammatory response^10,11^. PVL-associated diseases occur particularly in immunocompetent young patients who have previously been healthy. Clinically, they manifest as recurrent or chronic SSTIs or as abscessing/necrotizing pneumonia. This condition can complicate viral respiratory infections such as influenza and has a mortality rate approaching 40%^12^. In a central European setting, PVL genes can be expected to be present in approximately 1% of randomly tested *S. aureus* isolates. However, PVL is significantly more common in isolates obtained from SSTI^6^. There are also large geographic differences in prevalence, with PVL rates in Africa^13–16^ being much higher than in Central or Northern Europe.

In the 1930/40s and 1960s, as well as from the mid-1990s to the present day, there have been worldwide outbreaks of furunculosis caused by PVL-positive strains. The oldest known strains were susceptible to penicillin, and representative strains from this era, ATCC25923^17^ and “Oxford Staphylococcus”^18^, are still widely used as controls for susceptibility tests. The strains that dominated in the 1960s (phage type 80/81) and thereafter harboured penicillinase. Since the 1990s, PVL-positive MRSA strains gained considerable attention, starting with outbreaks in the USA and Australia. In the USA, one distinct PVL-positive MRSA strain, dubbed “USA300”, became extremely common, so that it was considered largely synonymous with “PVL-MRSA” or even with “community-associated” MRSA. However, this is misleading as other lineages dominate in other parts of the world, and as there are also community-associated MRSA that do not carry PVL genes. Recent studies have documented a continued global expansion of PVL-positive MRSA from the community into hospital/healthcare settings and the emergence of novel, occasionally multiresistant, PVL-MRSA lineages. This development is a major public health concern due to increasingly limited therapy options, and it highlights a need for regional molecular surveillance as especially visible in the Indian subcontinent^19–21^, the greater Middle East^22–24^ and Australia^25,26^.

The abovementioned “USA300” strain belongs to the widespread CC8 lineage^27^ but it is unique in carrying an SCC*mec* IVa element complemented by a copper resistance gene, an arginine metabolic element (“ACME”), a gene cluster encoding a transmembrane channel (*opp3B/C*) and genes encoding a zinc-containing alcohol dehydrogenase (*adhC*) and a spermidine N-acetyltransferase (*speG*)^28^. While the rapid proliferation of the “USA300” strain often has been attributed to these peculiar features, one should keep in mind that in other regions of the world, other PVL-MRSA strains lacking these genes were equally successful. This includes closely related, but ACME-negative strains from Latin America and Spain. These carry a mercury resistance operon, either on a plasmid (GenBank CP007658) or integrated, together with the copper resistance gene, into a SCC*mec* IVc element (SCC*mec* [IVc+CoMer], as in GenBank CP007670, CP007672)^29–31^.

While there is a huge amount of data, both epidemiological and genomic, on MRSA and PVL-MRSA from North America, Western Europe, Australia and, increasingly, from East Asia and the Arabian Gulf states, the knowledge of the general situation and of current trends and developments in Eastern Europe, Central Asia and Africa is comparatively poor. One country for which there is a particular dearth of information is Lithuania. A search of the genome database at https://www.bv-brc.org/ (accessed 2026, Jan 6th ) revealed just three *S. aureus* genome sequences (GenBank CTXR01, CTYA01, CTYC01) from Lithuania. These were isolates, collected in 1996, belonging to two different variants of ST239-MRSA-III, an ancient but pandemic clone of hospital-associated MRSA^32^.

A prospective case-cohort single-centre study^33^ from Kaunas (the second largest city of the country) on paediatric patients hospitalised from 2012 to 2015 showed a high rate of PVL-positives (51.5%), a MRSA rate of 7.0% and a rate of PVL-MRSA of 4.8%. A European multicentre study on paediatric bone and joint infections^34^ identified 12 PVL-positive isolates out of 85 cases collected across thirteen centres in Spain, Lithuania, Israel, Italy, Germany, Greece and Romania, and as much as eight of these twelve isolates originated from Lithuania. Another study^35^ on isolates collected in 2019 to 2021 in Vilnius (the capital and largest city of the country) identified PVL in 7.3% of isolates with MRSA being more commonly PVL-positive than MSSA (42.2%, 19/45 vs. 5.0%, 37/700; *p* < 0.0001). Neither study provided molecular typing data. Finally, four veterinary isolates were recently identified as ST398 livestock-associated MRSA^36^ indicating that this emerging strain also reached Lithuania.

Consequently, the aim of the present study was to type PVL-positive isolates from routine diagnostic procedures at a Vilnius laboratory. This was performed using a previously established DNA-microarray-based system which facilitated the detection of resistance genes, virulence markers and other genes of interest as well as an assignment to clonal complexes, strains and SCC*mec* types. Some isolates were, because of their epidemiological relevance (see below), also subjected to whole-genome sequencing using Oxford Nanopore technology (ONT).

## Materials and methods

### Hospital and ward characteristics

The study encompassed Panton-Valentine leukocidin-positive MRSA and methicillin-susceptible *S. aureus* (MSSA) isolates. These strains were collected from two healthcare institutions in Vilnius and from healthy community volunteers in Vilnius. The participating healthcare institutions shall not be named but just be referred to as Hospital 1 and Hospital 2 to preserve institutional confidentiality and patient privacy. *S. aureus* strains were primarily isolated from medical wards, surgery wards and intensive care units. Strains were collected consecutively over two separate intervals: from January 2018 to December 2019 and from January to December 2024.

Hospital 1 is a regional/municipal acute-care hospital with some 400 beds dedicated to acute care. It provides a comprehensive range of general, medical, and surgical services to the Vilnius region. Hospital 2 offers high-level surgical services, specializing in the areas of acute conditions, severe trauma and broad-profile clinical surgery. The hospital has approximately 700 beds. All consecutive adult patients (≥ 18 years) with *S. aureus* isolated from clinical specimens were included in this study. If multiple isolates were obtained from the same person, only the first isolate was included.

Hospital isolates were obtained from patients with clinically confirmed infections. The collected hospital specimens were subsequently assigned into five distinct groups based on the primary site of infection: skin and soft tissue, blood, respiratory tract, urine, and other specimens from sterile sites (including peritoneal fluid, synovial fluid, prosthetic material and cerebrospinal fluid).

Regarding ethics approval, see the statement in a separate paragraph below.

### Community participant recruitment and inclusion criteria

Community strains were isolated from nasal and pharyngeal swabs of participants recruited in the “Vilnius Healthy Community” cohort. This cohort comprised healthy adult volunteers recruited from non-medical public and private sector institutions in the city of Vilnius, Lithuania. The institutions were approached and, upon agreement, presentations were given to potential participants explaining the aim of the study, eligibility criteria and sampling procedure. Individuals who agreed to participate and met the study criteria were enrolled after providing written informed consent. Participants completed a questionnaire in the presence of a researcher, after which nasal and pharyngeal swabs were collected for the detection of *S. aureus* carriage. The inclusion criteria were as follows: participants had to be permanent residents of Vilnius, aged 18 years or older, not hospitalised within the previous three years, and not employed in healthcare. Regarding ethics approval, see the statement in a separate paragraph below.

### Phenotypic identification of isolates

All specimens were inoculated onto blood agar with 5% sheep blood (Graso Biotech, Poland) and the selective chromogenic media CHROMagar *Staph aureus* (Graso Biotech, Poland) and ChromID^®^ MRSA SMART (bioMérieux, France). All plates were aerobically incubated for 24–48 h at 35 ± 2 °C. Identification as *S. aureus* was confirmed using the MALDI-TOF VITEK^®^ MS PRIME Microbial Identification System (bioMérieux, France) according to the manufacturer’s direct colony spotting protocol. Briefly, bacterial colonies were spotted onto a VITEK^®^ MS PRIME target plate using a sterile plastic loop and overlaid with 1 µL of matrix solution. Spectra were acquired and analysed using the VITEK^®^ MS PRIME Knowledge Base (KB) v3.2 database, and identification results were reported by the instrument software.

### Phenotypic determination of MSSA/MRSA status

Phenotypic classification of *Staphylococcus aureus* isolates as MSSA or MRSA was performed by cefoxitin disk diffusion testing on Mueller-Hinton agar (Bio-Rad, France), in accordance with EUCAST 2024 guidelines^37^. Commercial cefoxitin disks containing 30 µg of cefoxitin (Bio-Rad, France) were used. Isolates were classified as MSSA or MRSA according to EUCAST interpretive criteria. For quality control, *Staphylococcus aureus* ATCC^®^ 29213 was included in each testing run, as recommended by EUCAST.

### PCR studies

*S. aureus* isolates were analysed for *mecA*,* mecC*, *lukF-PV* and 16S rRNA genes using in-house multiplex real-time PCR assays. The 16S rRNA gene served as an internal amplification control. Primer and probe sequences (Table 1) were designed using Vector NTI Advance™ software (Thermo Fisher Scientific, Waltham, MA, USA) for sequence alignment and the FastPCR online tool for in silico primer evaluation.

**Table 1:** Sequences of primers and detection hydrolysis probes.

Target gene	Detection primers and hydrolysis probes	
16S rRNA	Frrs_m	ACAGGATTAGATACCCTGGTAGTCC
	Rrrs_m	CGTTGCGGGACTTAACCCAAC
	Rrrs_Pr	Cy5/TCACRACACGAGCTGACGACAGCCA/BHQ2
*lukF-PV*	Pvl_F	TGGTTGGGATGTTGAAGCACA
	Pvl_R	TTGCAGCGTTTTGTTTTCGAG
	Pvl_P	HEX/TGCCAGTGTTATCCAGAGGTAACT/BHQ1
*mecA*	*mecA*_F	CTTTACGATAAAAAGCTCCAACATGA
	*mecA*_R	CTATTAATGTATGTGCGATTGTATTGC
	*mecA*_P	ROX/TGGCTATCGTGTCACAATCGTTGACGA/BHQ2
*mecC*	mecC_F	ACTAATGGTATGGAACGTGTAGT
	mecC_R	CCAACCTATTTGTCTTCCRGTTTC
	mecC_P	FAM/TTTT+TAATTCTGCTGTKCCAGATTTACC/BHQ1

For preparation of *S. aureus* lysates, isolates stored at − 80 °C were subcultured on Brain Heart Infusion agar and incubated aerobically at 35 ± 1 °C for 24 h. Approximately 2–3 colonies were transferred into 500 µL of sterile deionized water with a 1 µl loop and vortexed briefly to obtain a homogeneous suspension. The suspensions were heated at 100 °C for 10 min, cooled to room temperature for 5 min, and centrifuged at 6,000 rcf for 10 min. The clear supernatant containing bacterial DNA was transferred into sterile tubes and stored at − 80 °C until multiplex real-time PCR analysis.

Reactions were performed in a total volume of 15 µL containing 1 µL of *S. aureus* lysate. The reaction mixture consisted of 7.5 µL of 2× SensiMix™ II Probe (Bioline Reagents, UK), 200 nM of each primer (Biolegio, Netherlands) and 100 nM of each hydrolysis probe (Biolegio, Netherlands). Amplification was performed using a Rotor-Gene Q 5plex HRM thermal cycler (QIAGEN, Germany) with initial denaturation at 95 °C for 10 min followed by 40 cycles of 95 °C for 20 s and 55 °C for 60 s. PVL PCR results for individual isolates are provided in Supplementary File 1.

### Lateral flow assay for the detection of PVL

For the detection of lukF-PV in staphylococcal cultures, a rapid lateral flow assay (LFA; Vellap GmbH, Weimar, Germany; see https://www.vellap.shop/shop/PVL-Research-Use-Only-p780298571; accessed 3 February 2026)^38^ was used. *S. aureus* pure cultures were incubated overnight on Columbia blood agar at 37 °C. One loopful of culture material (approximately 1 µL) was suspended in 300 µL of buffer and briefly vortexed. The suspension was then centrifuged to sediment the cells (4,000 x g; 1 min). Subsequently, 100 µL of the supernatant were applied to the sample well of the test device, which was incubated at room temperature for 10 min.

Results were interpreted visually. The presence of both the test and control lines was considered positive, whereas the presence of the control line alone was interpreted as negative. Absence of the control line was regarded as invalid.

Results for individual isolates are provided in Supplementary File 1.

### Microarray-based molecular characterisation

Isolates were characterised using a DNA-microarray-based assay (INTER-ARRAY, Bad Langensalza, Germany). The microarrays, related protocols and methods as well as probe and primer sequences were previously described in detail^39–42^.

Briefly, *S. aureus* was cultured on Columbia Blood Agar and colonies were harvested after overnight incubation. The DNA extraction was performed with a combination of lytic enzymes together with buffers and spin columns from a commercial kit (Qiagen, Hilden, Germany). A multiplex linear amplification covering all target sequences was performed using one specific primer per target. During this step, biotin-16-dUTP labels were incorporated into the amplicons. After incubation and washing, hybridisation to the array-bound probes was performed. Streptavidin–horseradish peroxidase was then added, binding to the biotin labels incorporated into the amplicons. In the next step, the peroxidase triggered a localised precipitation of a dye at the positions where the specific amplicons were bound to the probes. Microarrays were photographed and analysed using a designated reader and software.

Results for the individual isolates are provided in Supplemental File 1.

### Whole-genome sequencing

Three isolates were subjected to whole-genome sequencing. These included two 2024 isolates from Hospital 2 that had been assigned by microarray to the presumptive outbreak strain. Their array profiles differed, indicating that one isolate, Vilnius-99243 carried a beta-haemolysin converting prophage (being positive for *sak*,* chp* and *scn*) while Vilnius-99272 lacked these genes. For comparison, the Lithuanian ACME- and PVL-positive “USA300” isolate Vilnius-99207, isolated in 2018 at Hospital 1, was also sequenced.

Whole genome sequencing by Oxford Nanopore Technology was performed using the MinION platform (Mk1B; Oxford Nanopore Technologies, ONT, Oxford, UK) as previously described^43–47^. In short, overnight cultures were grown at 37 °C on Columbia Blood Agar (Becton Dickinson GmbH, Heidelberg, Germany). The DNA extraction was performed using the NucleoSpin Microbial DNA kit (Macherey-Nagel, Düren, Germany) according to the manufacturer’s instructions. This was followed by an Agencourt AMPure XP (Beckman Coulter, Brea, CA, USA) purification step at a 1:1 ratio (v/v). The ONT ligation-native barcoding kit (SQK-NBD112.24) was used for the preparation of the library. This was done according to the protocol provided by ONT although incubation times for the A-overhang generation were modified for improved efficiency (10 min at 20 °C and 10 min at 65 °C). For library quantification, the Qubit 4 Fluorometer (ThermoFisher Scientific) was used. The initial flow cell check prior to sequencing identified approximately 1,500 active pores. Approximately 100 ng of DNA of each strain were loaded into the flow cells (FLO-MIN114).

Sequencing ran for 72 h using MinKNOW software (v25.02.16). Basecalling was performed with Dorado using model *dna_r10.4.1_e8.2_400bps_sup* and a minimum read Q-score threshold of 10. Barcoded reads were demultiplexed with Dorado using barcode detection at both read ends and exported as per-barcode FASTQ files. The per-barcode FASTQ files were subsequently filtered with Filtlong, retaining the best 90% of reads, with a target of 500 Mbp per barcode and a minimum mean read quality of Q15. Read-level quality control was performed at multiple stages. Overall sequencing-run quality metrics were generated from the full basecalled FASTQ file using NanoPlot. Per-barcode read quality and length distributions before and after Filtlong filtering were compared using NanoComp. In addition, FastQC was run for all per-barcode FASTQ files, and the resulting reports were summarized using MultiQC. To assess potential barcode carry-over, the pipeline scanned full-length FASTQ reads and polished FASTA assemblies for exact matches to the forward and reverse NB01–NB24 barcode sequences, producing separate barcode-contamination tables for read and assembly files. Genome assemblies were generated with Flye (v2.9.1) using the *--nano-hq* and *--meta *options. Assembly statistics were obtained from the combined assembly summary generated by our in-house pipeline. Assembly graph visualizations were produced with Bandage from the Flye GFA files. The resulting draft assemblies were subsequently corrected in two polishing stages. First, the filtered nanopore reads were aligned against the corresponding draft assembly using minimap2. Four iterative rounds of Racon (v1.5.0) polishing were then performed, with the output of each round serving as the reference for the following round. Racon was run with match, mismatch and gap scores of 8, − 6 and − 8, respectively, and a window length of 500 bp. In the second polishing stage, the assembly obtained after the fourth Racon iteration was further corrected with Medaka (v2.1.0) using the model *r1041_e82_400bps_sup_v5.2.0*.

The sequences of the three isolates are provided in Supplemental File 2 and they were submitted to GenBank, BioProject PRJNA1444031; BioSample accession numbers SAMN56737420, SAMN56737421, SAMN56737422.

### cgMLST analysis

**Fig. 1:**
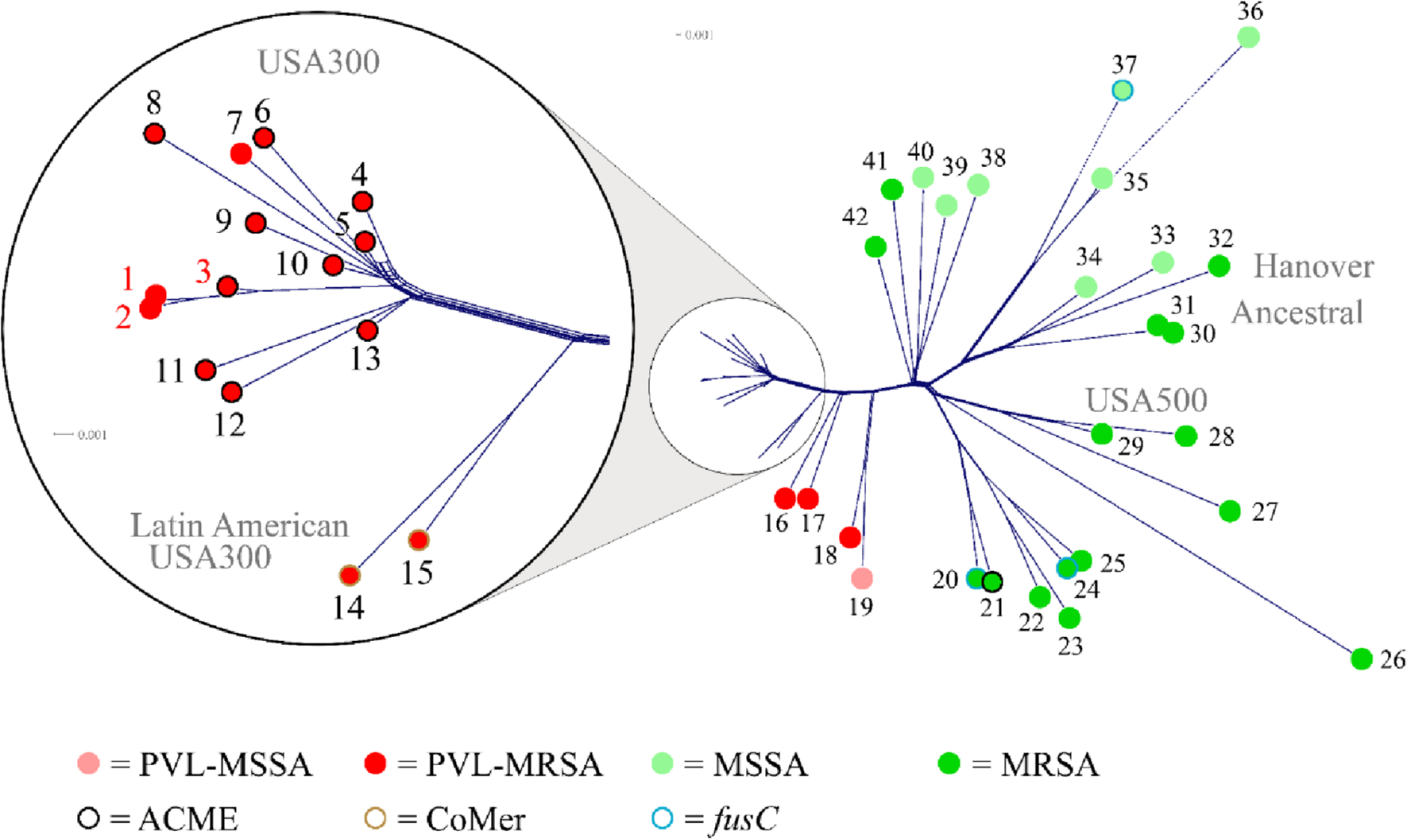
SplitsTree analysis of 42 CC8 genomes. The branches of “USA300” and Latin American “USA300-like” strains are shown as enlarged insert. The numbers match those in the first column of Table 2. Study strains are indicated in red font. The first row of the legend indicates the colour codes of the *mecA* and PVL status, the second one, accessory SCC elements (ACME, copper/mercury resistance, fusidic acid *fusC* resistance).

For comparison and phylogenetic analyses, 39 additional high-quality genomes of *S. aureus*, including MSSA and MRSA with different SCC*mec* elements, were downloaded from GenBank (for accession numbers, see Table 2). For these, as well as for the three genomes from the present study, core genome multilocus sequence typing (cgMLST) was performed using the online tool at https://pubmlst.org/bigsdb?db=pubmlst_saureus_seqdef&page=sequenceQuery (see also^48^; accessed in Dec. 2025/Jan. 2026). All cgMLST markers that yielded identical alleles in all 42 genomes were excluded from the analysis. The remaining 1,761 out of 2,215 genes were further analysed. Then, genes were excluded if they were absent in any of the 42 genomes, truncated or interrupted in one or more genomes, or exhibited divergent lengths. Such variations are commonly attributable to mutations, integration of mobile genetic elements or sequencing errors. The remaining 1248 gene sequences for each of the 42 genomes were, if necessary, changed into forward (5´to 3´) orientation (Supplemental file 3a) and concatenated, resulting in 42 sequences á 1,248,306 bases each (Supplemental file 3b). Because of the limitations to the computing power of a personal computer, all bases were removed that were identical at their respective positions of all 42 genomes. Thus, a string of 3,754 nucleotides remained representing all single nucleotide polymorphisms occurring at least once in the 1,248 target genes of the 42 strains (Supplemental file 3c). These strings or “sequences” were processed using SplitsTree 4^49^ on default settings, resulting in the *.nex file provided in Supplemental file 3d. The resulting graph was slightly edited and enhanced with regard to colours, resolution and font size to yield a printable and legible figure (Fig. 1).

**Table 2:** CC8 genomes that were used for comparison and cgMLST analysis.

No.	Strain affiliation	GenBank accession	cgMLST type	Strain ID	Geographic origin
**1**	ST8-MRSA-IVa (PVL+)	*	4486, 7694, 18773, 23994, 27813, 27814, 27934, 30198, 30199; all with 54 mm	Vilnius-99243	Lithuania
**2**	ST8-MRSA-IVa (PVL+)	*	4486, 7694, 18773, 23994, 27813, 27814, 27934, 30198, 30199; all with 54 mm	Vilnius-99272	Lithuania
**3**	ST8-MRSA-[IVa+ACME1] (PVL+), “USA300”	*	4486, 7694, 18773, 23994, 27813, 27814, 27934, 30198, 30199; all with 44 mm	Vilnius-99207	Lithuania
**4**	ST8-MRSA-[IVa+ACME1] (PVL+), “USA300”	CP000255	20829	FPR3757	USA, CA
**5**	ST8-MRSA-[IVa+ACME1] (PVL+), “USA300”	CP019590	27854	C2406	Canada, AB
**6**	ST8-MRSA-[IVa+ACME1] (PVL+), “USA300”	CP007176	27807	USA300-ISMMS1	USA, NY
**7**	ST8-MRSA-IVa (PVL+)	LR130518	28478	BPH2986	Australia
**8**	ST8-MRSA-[IVa+ACME1] (PVL+), “USA300”	CP051907	18773, 27934, 30198, 30199; all with 71 mm	ER11450-3	USA, NY
**9**	ST8-MRSA-[IVa+ACME1] (PVL+), “USA300”	CP013231	27834	UTSW MRSA 55	USA, TX
**10**	ST8-MRSA-[IVa+ACME1] (PVL+), “USA300”	CP010300	27815	BAA-1680 27b	USA, TX
**11**	ST8-MRSA-[IVa+ACME1] (PVL+), “USA300”	CP151123	10633, 11050, 16390; all with 45 mm	BSN46-2	USA, MI
**12**	ST8-MRSA-[IVa+ACME1] (PVL+), “USA300”	CP165650	16390 and 17634; all with 33 mm	B0354	USA, CA
**13**	ST8-MRSA-[IVa+ACME1] (PVL+), “USA300”	CP000730	21483	TCH1516	USA, TX
**14**	ST8-MRSA-[IVc+CoMer] (PVL+), “Latin Am. USA300”	CP007672	27822	CA12	Colombia
**15**	ST8-MRSA-[IVc+CoMer] (PVL+), “Latin Am. USA300”	CP007670	27819	M121	Colombia
**16**	ST8-MRSA-IVa (PVL+)	CP007674	27820	CA15	Colombia
**17**	ST8-MRSA-IVc (PVL+)	CP007676	22774	HUV05	Colombia
**18**	ST923-MRSA-IVa (PVL+)	CP007657	2221 with 22 mm	V2200	Venezuela
**19**	ST8-MSSA (PVL+)	CP127017	28339	TT-2020-042	Trinidad & Tobago
**20**	ST8-MRSA-[IV+*fusC*]	LT671859	28430	Giessen isolate-I	Germany
**21**	ST8-MRSA-[IV+ACME2]	HF937103	21796	M1	Denmark
**22**	ST8-MRSA-IVF, “Irish AR43/Irish-02”	LS483316	28462	NCTC13395	UK
**23**	ST8-MRSA-IID, “Irish AR13/14”	LS483301	28455	NCTC13394	UK
**24**	ST8-MRSA-[VI+*fusC*]	LS483319	28461	NCTC13140	UK
**25**	ST8-MRSA-IVh/j, “UK-EMRSA-2”	LR134085	28480	NCTC12233	UK
**26**	ST1516-MRSA-IVc	CP030137	22196 or 39684	M51	China, Guangdong
**27**	ST113-MRSA-IVc	CP039157	16063 with 170 mm	P10	Pakistan
**28**	ST612-MRSA-IVb/d/i	CP029166	27889, 35401, 35402 or 39671	SVH7513	Australia, NSW
**29**	ST8-MRSA-IVb/d/i, “USA500”	CP007499	3945 or 3988; both with 30 mm	Strain 2395	USA
**30**	ST254-MRSA-I	CP000046	21467	COL	UK
**31**	ST250-MRSA-I	CP007539	27808	NRS100 = FDAARGOS-5	US, NY
**32**	ST254-MRSA-PseudoSCCmec, “Hanover Epidemic Strain”	CP022902	12235 with 60 mm	Strain 165	Germany
**33**	ST254-MSSA	AP009351	20401	Newman	Laboratory strain
**34**	ST6271-MSSA	CP023500	31567	FDAARGOS-412	USA
**35**	ST8-MSSA	CP000253	21478	NCTC8325	UK
**36**	ST8-MSSA	CP003033	21482	RN4220VC40	Laboratory strain
**37**	ST8-MSSA-*fusC*	CP077933	28098 with 177 mm	Augsburg strain 359	Germany
**38**	ST8-MSSA	CP123745	28393, 39791	Muenster strain 1–7	Germany
**39**	ST8-MSSA	CP022892	27909	Muenster strain 54	Germany
**40**	ST8-MSSA	CP022290	27877	EDCC5458	Germany
**41**	ST8-MRSA-IVc	AP017377	27868	OC8	Russia, Sibiria
**42**	ST8-MRSA-IVb/d/i	CP103850	28266, 29198	NRL02-947	Czech Republic

* Submitted to GenBank, release at the time of manuscript submission pending, see also Supplemental file 2. mm=mismatches.

## Results

### PVL detection by PCR, microarray and LFA

Out of 1,296 *S. aureus* isolates (1,170 MSSA and 126 MRSA), 124 yielded PVL-positive PCR results. 100 isolates were available for genotyping. For two of the 100 isolates, both methicillin-susceptible and community-associated, PVL could not be confirmed, neither by microarray nor by LFA. A concordance of PVL detection by microarray and LFA was observed in 100/100 isolates (100%). All strains identified phenotypically as MRSA were genotypically confirmed as *mecA-*positive. The MRSA rate among the PVL-positive isolates was 61.2% (60 out of 98).

In addition, seven PVL-negative MSSA (CC5, CC45, CC97, CC101, CC398, CC2867) were isolated when re-cloning PVL-MRSA prior to genotyping. These were regarded as contaminants and are not discussed further, but they are listed in Supplemental File 1.

### Clonal complex and strain affiliations

Clonal complex and strain affiliations of the isolates from the community and the two hospitals are shown in Table 3. The underlying microarray data are provided in Supplemental File 1.

**Table 3:** Clonal complexes and strains from the three sampling sites.

Clonal Complex	Strain	Community (*n*)	Community (%)	Hospital 1 (*n*)	Hospital 1 (%)	Hospital 2 (*n*)	Hospital 2 (%)
CC8	CC8-MRSA-[IV+ACME] (PVL+), “USA300”	0	0.00	11	25.00	3	6.38
	ACME-negative CC8-MRSA-IV (PVL+)	0	0.00	1	2.27	42	89.36
CC22	CC22-MSSA (PVL+)	1	11.11	0	0.00	0	0.00
	CC22-MRSA-IV (PVL+/*tst+*)	0	0.00	0	0.00	1	2.13
CC25	CC25-MSSA (PVL+)	0	0.00	1	2.27	0	0.00
CC30	CC30-MSSA (PVL+)	2	22.22	13	29.55	0	0.00
CC45	CC45-MSSA (PVL+)	0	0.00	1	2.27	0	0.00
CC59	CC59-MSSA	1	11.11	0	0.00	0	0.00
	CC59-MSSA (*ccrC*/PVL+)	0	0.00	1	2.27	0	0.00
	CC59-MRSA-VT *(ermB+/aphA3+/*PVL+), “Taiwan Clone”	0	0.00	1	2.27	0	0.00
CC80	CC80-MRSA-IV (PVL+)	0	0.00	0	0.00	1	2.13
CC88	CC88-MSSA (PVL+)	0	0.00	1	2.27	0	0.00
CC121	CC121-MSSA (PVL+)	4	44.44	12	27.27	0	0.00
CC152	CC152-MSSA (PVL+)	0	0.00	1	2.27	0	0.00
CC361	CC361-MSSA (PVL+)	0	0.00	1	2.27	0	0.00
CC398	CC398-MSSA	1	11.11	0	0.00	0	0.00

In the community, CC121-MSSA (PVL+) and CC30-MSSA (PVL+) were the most frequently detected strains, although overall isolate numbers were low. This pattern was corroborated in Hospital 1, where CC30-MSSA (*n* = 13; 29.6%) and CC121-MSSA (*n* = 12; 27.3%) were likewise frequently detected. Eleven isolates from Hospital 1 (25.0%) were assigned to CC8-MRSA-[IV+ACME] (PVL+) “USA300”. A markedly different distribution was observed in Hospital 2. In total, 42 isolates (89.4%) belonged to a single clone, a PVL-positive, ACME-negative CC8-MRSA-IV. Five additional isolates were assigned to three other PVL-MRSA strains (CC8-MRSA-[IV+ACME] (PVL+) “USA300”, CC22-MRSA-IV (PVL+/*tst*+) and CC80-MRSA-IV (PVL+)), which are also known from other regions worldwide (see Discussion).

### Description of the PVL-positive, ACME-negative CC8-MRSA-IV outbreak strain

Two isolates of the PVL-positive, ACME-negative CC8-MRSA-IV outbreak strain from Hospital 2 were sequenced (Supplemental file 2). Both belonged to ST8 and RIDOM *spa* type t051 (repeat succession 11-19-21-12-21-17-34-24-34-22-25; Kreiswirth nomenclature Y1-H1-F1-G1-F1-M1-B1-Q1-B1-L1-O1).

Both isolates carried an SCC*mec* IVa element with the *dru* type dt9g (5a-4a-0-2d-5b-3a-2 g-3b-4e) which is the type commonly associated with the “USA300” strain^50,51^. However, the entire set of “USA300”-specific genes downstream of the SCC*mec* IVa element (corresponding to USA300HOU_0049 to 0085 in GenBank CP000730.1, including ACME, *opp3B/C*,* speG*,* adhC*) was absent. Thus, the *copA2-*SCC gene was also absent, indicating a difference compared to both, “USA300” and Latin American/Spanish “USA300-like” CC8-MRSA-IV. The mercury resistance operon was neither identified in the genomes nor in the plasmid sequence of Vilnius-99243 and Vilnius-99272, and it was also not found by microarray analyses of all 43 isolates.

In Vilnius-99243 and Vilnius-99272, genes *cadD/X* (cadmium resistance), *mpbBM+msrA* (macrolide resistance), *aadE*_truncated_+*sat+aphA3* (aminoglycoside and streptothricin resistances) as well as the penicillinase-operon *blaZ/I/R* were located on one single plasmid. This plasmid was nearly identical to those in the “USA300” strains TCH1516 and USA300-ISMMS1 (pUSA300HOUMR, GenBank: CP000731.1 and pUSA01-ISMMS, GenBank: CP007177.1) differing only in an insertion of *qacAB* and *qacR*, a gene encoding a quaternary ammonium compound resistance protein and its regulator (Supplemental File 4).

Vilnius-99243 carried three prophages, Vilnius-99272 had two, lacking the *hlb*-integrating phage. One prophage was a A5IT17-integrating *Triavirus* prophage that carried PVL genes and a MW1442-like phage integrase. Its gene content was identical to the one of the corresponding prophages in “USA300” sequences (CP000255, CP000730, CP007176, CP010300, CP013231, CP051907, CP151123, CP165650) as well as to those in Latin American, ACME-negative “USA300-like” sequences (CP007674, CP007657, CP007670, CP007672, CP007676) and to Caribbean PVL-MSSA (CP127017, CP127025). However, the PVL-prophages of the outbreak isolates Vilnius-99243 and Vilnius-99,272 differed from all these sequences in a total of three SNPs (positions 13,865, 25,947, 40,226 in Supplemental File 5).

Furthermore, Vilnius-99243 contained a *Dubowvirus*-like prophage sequence integrated into A5IU43 = *yfkAB* (SAUR2054). In contrast to the previously mentioned prophage, the carriage of a phage in this position was variable, with several “USA300” (CP000255, CP000730, CP010300, CP013231) and ACME-negative “USA300-like” sequences (CP007674, CP007657, CP007672, CP007676) lacking a phage insertion at this site. The A5IU43 = *yfkAB-*integrating prophage sequences of the strains that carried such a prophage (CP007176, CP051907, CP151123, CP165650, CP007670 and Vilnius-99243) all differed in gene content and size with no two of them being completely identical (Supplemental File 6).

A third prophage integration site was *hlb*, the haemolysin beta gene. Thirty-eight out of 43 Lithuanian isolates carried, as shown by microarray, *sak*,* scn* and *chp*.

These genes, encoding staphylokinase, staphylococcal complement inhibitor and chemotaxis-inhibiting protein, are associated with an *hlb*-integrating *Biseptimavirus* prophage. This prophage was highly conserved (Supplemental File 7). Sequences in Vilnius-99243 and in all “USA300” and “USA300-like” strains (CP000255, CP000730, CP007176, CP010300, CP013231, CP051907, CP151123, CP165650, CP007674, CP007670, CP007672, CP007676) were essentially identical, differing only in a low number of SNPs and/or gaps that may be attributable to sequencing errors. One reference sequence had a divergent prophage (CP007657).

Vilnius-99272 lacked that phage, and according to microarray analyses, four other Hospital 2 isolates also did so.

Regarding other potentially mobile genetic elements, most isolates and sequences of “USA300” and Latin American/Spanish lineage of “USA300-like” CC8-MRSA-IV harbour a pathogenicity island that includes enterotoxin genes *sek* and *seq.* These were absent from the Vilnius-99,243 and -99,272 genome sequences as well as, according to microarray experiments, from all 43 isolates.

A conspicuous feature of all isolates was the absence in microarray analyses of a signal for *bbp* (also known as *sdrE*), encoding bone sialoprotein-binding protein E. The analysis of the genome sequences of Vilnius-99243 and -99272 revealed a deletion of a part of the downstream, 3’-part of the previous gene, *sdrD* (serine aspartate repeat protein D, bone sialoprotein-binding protein), its rho-independent terminator sequence and of the 5’-part of *bbp=sdrE.* This resulted in a 4014 bp fusion product of *sdrD* and *bbp* in which a major 5’-part was identical to *sdrD* in “USA300” reference sequences (CP000730, CP010300) while a minor 3’-part was identical to their *bbp=sdrE* (see Supplemental File 8). A very similar phenomenon is also present in the ST8-MSSA reference sequence NCTC8325, GenBank CP000253.1 (locus tag SAOUHSC_00545) although the exact size and boundaries of the deleted region differ. Two “USA300” sequences from the US (CP031888, CP031890) also showed such a *sdrD/bbp* fusion, but size and boundaries of the deleted region differed from both, the Vilnius sequences and from NCTC8325 (see Supplemental File 8).

### Comparison of the outbreak strain to Lithuanian “USA300”

Twelve isolates, eleven from Hospital 1 and one from Hospital 2, were assigned by microarray to “USA300”, i.e., to ACME- and PVL-positive CC8-MRSA-IV. One isolate, Vilnius-99207, was sequenced by ONT (Supplemental file 2), revealing a presence of an SCC*mec* IVa+ACME1 element including *adhC*,* speG* and *copA*2-SCC. It was assigned to ST8, RIDOM *spa* type t051 (11-19-21-12-21-17-34-24-34-22-25) and to *dru* type dt9g (5a-4a-0-2d-5b-3a-2 g-3b-4e).

The plasmid in Vilnius-99207 was identical to the one in TCH1516 and USA300-ISMMS1 (pUSA300HOUMR, GenBank: CP000731.1 and pUSA01-ISMMS, GenBank: CP007177.1). Accordingly, it differed from the plasmids of Vilnius-99243 and -99272 by lacking *qacAB* and *qacR*. Indeed, an absence of *qacAB* was noted by microarray for all “USA300” isolates from this study, while it was present in 42 out of 43 isolates of the presumptive outbreak strain.

The PVL-prophage of the “USA300” isolate Vilnius-99207 was, with regard to gene content, identical to all reference sequences analysed (see above). Two of the characteristic SNPs of Vilnius-99243 and Vilnius-99272 (pos. 13,865 and 40,226) were also observed in Vilnius-99207 whereas at position 25,947 its sequence matched those of the previously published strains (Supplemental File 5).

In this strain, there was no *Dubowvirus-*like prophage sequence integrated into A5IU43 = *yfkAB*. The haemolysin-beta-converting phage was identical as in other sequences of ST8 MRSA (see above and Supplemental File 7).

The deletion of the 3’-part of the *sdrD* gene and the 5’-part of *bbp=sdrE* was also observed in the genome sequence of the Lithuanian “USA300” isolate Vilnius-99207 (Supplemental File 8) that was *bbp*-negative according to microarray, as were the other eleven “USA300” isolates from the present study.

### cgMLST of the outbreak strain, Lithuanian “USA300” and of other CC8 genomes

The analysis was based on 3,754 SNPs in 1,248 cgMLST genes representing a total of 1,248,306 bases per strain, as described in the Materials and Method section and in Supplemental Files 3a to 3d. A graphical representation is shown in Fig. 1.

The two Vilnius outbreak strain sequences, Vilnius-99243 and Vilnius-99272, differed by 13 SNPs. The most similar sequence was the Lithuanian “USA300” isolate Vilnius-99207, differing by 22 and 23 SNPs, respectively. The other nine “USA300” sequences considered (for IDs and accession numbers, see Table 2) differed by an average of 71.5 SNPs, with a range of 54 to 90 SNPs for the individual sequences. Interestingly, the Australian PVL-positive ST8-MRSA-IVa isolate BPH2986 (GenBank LR130518) also laid in this range indicating it also to be a variant of “USA300” that similarly lost ACME and accompanying genes. In contrast to the Lithuanian strain, however, it carries enterotoxin genes *sek* and *seq*, and it lacks the truncation of the *bbp* gene.

Other PVL-MRSA sequences carrying plain SCC*mec* IVa elements were markedly more distant, with 164 and 165 SNP differences for CA15 (CP007674) and 204 and 205 SNP differences for V2200 (CP007657).

Additional observations (Fig. 1) included the close relationship of Latin American ST8-MRSA-[IVc+CoMer] to “USA300”, as previously reported^27^, and of the epidemic Trinidad PVL-MSSA (GenBank CP127017;^52^ to CoMer-negative, PVL-positive ST8-MRSA from Latin America.

## Discussion

The high MRSA rate (61.2%) among PVL-positive *S. aureus* isolates indicates a concerning convergence of virulence and resistance. In Germany, a comparable MRSA rate among PVL-positive isolates (56%) was reported^38^ following a steep increase of PVL-MRSA at the expense of PVL-MSSA compared with the preceding decade^53^. For Lithuania, no such comparison is possible and the general epidemiological situation of PVL-positive *S. aureus*, and *S. aureus* in general, remains relatively obscure due to the lack of molecular typing data.

The marked predominance of CC8-MRSA-IV in one hospital strongly suggests a local outbreak. In contrast, the diversity of clonal complexes observed in the other hospital and in the community indicates polyclonal, community-associated transmission and possible importation of internationally circulating lineages, as described elsewhere in Europe.

Eight strains of PVL-MSSA and one of a PVL-negative MSSA were identified with one isolate each. Two strains (PVL-positive CC88-MSSA, CC152-MSSA) are known to be associated with origin from, or travel to, Africa. CC361-MSSA are frequently found in North Africa and the Middle East although they are typically PVL-negative. PVL-positive CC45 isolates are extremely rare (author´s unpubl. observation) so its detection in a rather small sample was surprising. Another isolate of that strain was previously found by the authors in Romania^54^. Its abundance and distribution in Eastern Europe are yet to be investigated, underscoring the very limited knowledge regarding the molecular epidemiology of MSSA in large parts of Europe. Two PVL-negative MSSA isolates (CC59, CC398) were identified. These lineages also include PVL-positives so that it cannot be determined whether these isolates were false-positive in the PCR or if they lost the PVL-prophage during freezing, thawing and subculturing.

Three distinct strains of PVL-MRSA were represented by one isolate each. This included CC22-MRSA-IV (PVL+/*tst+*). This is an emerging, potentially pandemic, strain that apparently was found first in Iran^55^ and that spread thereafter across Arabian Gulf countries^23,56^, Nepal^57^ and Pakistan (GenBank CP038850) all the way to China^58^ and Japan^59^. Another isolate was assigned to CC59-MRSA-VT (*ermB+/aphA3*+/PVL+), a strain known as “Taiwan Clone” from South-East Asia^60,61^ which, meanwhile, has also been found in neighbouring Poland^62^. Finally, CC80-MRSA-IV (PVL+) is a sporadic CA-MRSA mostly known from, and associated with travel to, Mediterranean or Middle Eastern countries^63–66^.

Four strains, two MSSA and two MRSA strains were common, yielding much more than just single or sporadic isolates. These MSSA strains included CC30-MSSA (PVL+) with 15 isolates, from the community and Hospital 1. This is an ancient pandemic lineage that frequently carries PVL, and PVL-positive isolates belonging to this clonal complex have been collected already as much as 80 years ago^17,18,67^. Similarly, CC121-MSSA (PVL+), with 16 isolates from the community and Hospital 1, is known to occur pandemically in most diverse geographic settings^67–71^.

With a total of fourteen isolates, CC8-MRSA-[IV+ACME] (PVL+), “USA300” was the fourth most common strain. As mentioned above, this strain emerged to be extremely common in the United States. It is also common or epidemic in Canada, Cuba and other Caribbean islands, Suriname, Romania, in the Gulf Emirates, Pakistan/Afghanistan and Australia^72–77^. Elsewhere, including in parts of continental Western Europe, it is sporadically found, and many of the cases detected have a travel history^42,78–80^. Its high prevalence in Lithuania, however, is not surprising. After political suppression in Tsarist Russia and the Soviet invasion, a large Lithuanian diaspora developed which comprises half as many people (1.4 million; https://en.wikipedia.org/wiki/Lithuanians; accessed at Jan., 7th 2026) as the population of Lithuania (2.8 million). Half of the Lithuanian diaspora lives in the USA (about 711.000) making Chicago the sixth largest “Lithuanian” city (with about 60,000 Lithuanians and/or people of Lithuanian descent; https://en.wikipedia.org/wiki/Lithuanians_in_the_Chicago_area; accessed at Jan., 7th 2026) after Vilnius, Kaunas, Klaipėda, Šiauliai and Panevėžys. After Lithuania re-gained its independence, a lot of travel took place in either direction, so that an importation of an American epidemic MRSA strain into Lithuania appears to be highly probable.

The most prevalent strain was, strikingly, an ACME-negative CC8-MRSA-IV (PVL+). The strain appears to have emerged from “USA300” rather than from any other CC8 sub-lineage. As shown, “USA300” exists in Lithuania, with Hospital 1 isolates dating back to 2018. The Lithuanian “USA300” isolate sequenced in the present study indicated both, a close genetic relationship to the Vilnius outbreak strain, and to all other “USA300” sequences analysed. It shared with the Vilnius outbreak strain a unique deletion of parts of *sdrD* and *bbp* as well as two out of three SNPs identified in the PVL prophage. However, it had the same plasmid as North American “USA300” that differed from the outbreak strain in absence of *qacA/B*.

Taken together, the available evidence suggests that the North American epidemic strain CC8-MRSA-[IV+ACME] (PVL+), “USA300” was imported into Lithuania prior to or in 2018 (when Vilnius-99207 was collected). From the “USA300” strain, particularly from a variant with a distinctive *sdrD/bbp* deletion, a novel clone emerged by (i) the loss of the ACME, *opp3B/C*,* speG*,* adhC* and *copA2-*SCC genes, by (ii) a mutation leading to the third SNP in the PVL prophage in addition to the two SNPs already distinguishing the Lithuanian from other “USA300” sequences and by (iii) acquisition and integration of *qacAB* and *qacR* genes into the multi-drug resistance plasmid originally carried by the strain. Whether this emergence occurred in Lithuania or overseas, cannot be deduced, unless, possibly, more genome sequences were released.

This episode also shows that a “USA300”-derived strain without the ACME element and its accompanying genes is still a formidable pathogen that is able to cause a major outbreak. This finding parallels the observations from Latin America, were other ACME-negative, PVL-positive CC8-MRSA prevailed.

The predominance of this strain in Hospital 2 strongly suggests an outbreak situation. Follow-up investigations in this hospital, in cooperating healthcare facilities (e.g., rehabilitation units and outpatient clinics), and across the Vilnius municipality are urgently needed. This raises the question of how to rapidly detect and trace such outbreaks.

Rapid PVL detection is essential for preventing outbreaks of strains such as “USA300” and the Vilnius outbreak clone, and for improving clinical outcomes. Especially in settings with limited epidemiological data, such as Lithuania, molecular typing, rapid screening and strengthened infection control are critical. Given its clinical relevance, routine PVL screening—via PCR or LFA—should be seriously considered in diagnostic practice.

From an economic perspective, lateral flow assays are the most accessible option for PVL screening, costing only a few euros per test and providing results within approximately 15 min. This makes them particularly suitable for resource-limited settings and decentralised laboratories serving smaller hospitals with low sample volumes. PCR, as described above, is also a convenient tool, particularly within a centralised laboratory structure serving multiple hospitals, when higher number of samples are to be screened. DNA microarray typing allows rapid, parallelised clonal complex and SCC*mec* profiling at much lower cost than whole-genome sequencing. Microarray-based typing provides rapid and comprehensive profiling of clonal complexes, SCC*mec* types, and virulence and resistance genes. Long-read sequencing enables complete assembly of prophages, SCC elements and plasmids, which is critical for detailed strain characterisation, whereas short-read sequencing may offer higher precision for SNP detection.

While whole-genome sequencing is indispensable for outbreak phylogenomics, it remains too expensive for standard diagnostic labs. Submission of isolates to centralised reference laboratories often results in time lags of days to weeks^81^, limiting the immediate relevance of WGS results for patient management and infection control. A tiered diagnostic approach—using LFAs or PCR for routine screening, microarrays for real-time outbreak investigations, and whole-genome sequencing for retrospective confirmation or special cases such as forensic investigations or characterisation of novel strains—appears to be a practical strategy.

Beyond diagnostic screening, public health strategies should include a mandatory notification of PVL-MRSA to public health authorities. In the German State of Saxony, this is already implemented^82,83^. As cases frequently appear in clusters, including family members, colleagues or fellow members of sports teams^84^, fire-fighters^85^ or military units^86^, contact tracing of PVL cases (regardless of methicillin resistance or susceptibility) is urgently recommended. Given that sporadic cases may be travel-associated, patients with suspected PVL-associated disease should be asked for a history of travel or migration. In case of hospitalisation, they also should be treated as MRSA cases until proven otherwise, including pre-emptive isolation. Beyond that, decolonisation measures for any confirmed carriers of PVL-positive *S. aureus* and their close contacts should be considered^87^.

In conclusion, we describe a high rate of MRSA among PVL-positive clinical and surveillance isolates from Vilnius, Lithuania. While the methicillin-susceptible PVL-positive isolates belong to well-known and wide-spread lineages (CC30 and CC121), the methicillin-resistant ones belong to the “USA300” clone, most likely imported from North America, and to a locally evolved variant of this strain that lost the ACME element and accompanying genes. The virtual absence of genotyping data led to the unfortunate situation that an outbreak situation in one of the nation´s major hospitals went undetected. This emphasises the need for a genotypic surveillance of multiresistant and virulent bacterial pathogens.

## Supplementary Information

Below is the link to the electronic supplementary material.

Supplementary Material 1

## Data Availability

All relevant information is present in the manuscript and in the Supplemental Files, which also include the genome sequences. Sequences have been submitted to GenBank, BioProject PRJNA1444031; BioSample accession numbers SAMN56737420, SAMN56737421, SAMN56737422.
