# Supplementary material for: Molecular typing of PVL-positive Staphylococcus aureus isolates from Lithuania and genome sequencing of a local outbreak strain
Source: Sci Rep. 2026 Jul 18;16:22540. doi: 10.1038/s41598-026-61973-x (PMC13380623; doi:10.1038/s41598-026-61973-x)
Supplement: Supplementary file 1 — Supplementary Material 1 [file 41598_2026_61973_MOESM1_ESM.zip › Supplemental file 2b_Sequencing statistics.docx]

**Supplemental file 2b:** Statistics regarding ONT sequencing of the study strains.

| **Isolate ID** | **Assembly Length** | **Coverage** | **N50** | **Number of reads** |
| --- | --- | --- | --- | --- |
| **Vilnius-99207** | 2,862,910 | 134 | 12,025 | 48,793 |
| **Vilnius-99243** | 2,875,960 | 171 | 9,215 | 35,111 |
| **Vilnius-99272** | 2,848,625 | 147 | 10,873 | 48,186 |
